# The Exchange Rate of the Thiol Ligand of iPr_3_PAuSATg With the Cys-34 Residue of Bovine Serum Albumin

**DOI:** 10.1155/MBD.1994.520

**Published:** 1994

**Authors:** J. Xiao, C. Frank Shaw III

**Affiliations:** 1 Department of Chemistry University of Wisconsin-Milwaukee Milwaukee WI 53201-0413 USA


					THE EXCHANGE RATE OF THE THIOL LIGAND OF iPr3PAuSATg
WITH THE CYS-34 RESIDUE OF BOVINE SERUM ALBUMIN
J. Xiao and C. Frank Shaw III
Department of Chemistry, University of Wisconsin-Milwaukee, Milwaukee, Wl-53201-0413, USA
Auranofin [Et3PAuSATg=triethylphosphine-(2,3,4,6-tetra-O-acetyl-l-thio-8-D-glucopyranosato-S)-
gold(I)] is an antiarthritic drug. Its metabolism Includes two Iigand displacements at gold(I). A
reversible displacement of the thiol ligand of the drug by the Cys-34 residue of serum albumin is
observed as a first step in the in vitro reactions of the drug and albumin. This reversible feature may
facilitate gold(I) transfer from site to site in vivo. To investigate the rate of this thiol ligand exchange,
a 31 p NMR saturation transfer study was performed. In order to minimize the side reaction in which
phosphine is displaced and oxidized, iPr3PAuSATg [iPr3=tri(iso)propyl was used instead of
auranofin:
k
AIbSH + iPrPAuSATg , AIbSAuPiPr3
+ HSATg
The results show that the forward (kf= 0.12X10"3 M'ls"1) and reverse (kr= 0.39X10"4 M"ls"1) rates
occur at a similar order of magnitude. As expected, since the protein thiol, Cys-34, has a higher
affinity than the tetmacetyl thioglucose for gold(I), the forward reaction is more rapid than the
reverse reaction. The equilibrium constant for the reaction calculated from the rate constants, Keq
3.1, is smaller than expected for the nearly complete transfer of gold(I) from the thioglucose to the
Cys-34. The equilibrium constant of the reversible reaction was also measured by a liquid
chromatography method, which gave keq 3.0. This value is consistent with that derived from the
kinetic constants. An explanation for this unexpectedly small equilibrium constant is that the gold
binding to albumin is driven by further reactions of the displaced acetyl thioglucose.
520

				

